# Endogenous Protective Factors and Potential Therapeutic Agents for Diabetes-Associated Atherosclerosis

**DOI:** 10.3389/fendo.2022.821028

**Published:** 2022-04-26

**Authors:** Chaoqun Wang, Jin Chen, Pin Wang, Shengli Qing, Wenwen Li, Jin Lu

**Affiliations:** ^1^ Department of Endocrinology and Metabolism, The First Affiliated Hospital of Naval Medical University, Shanghai, China; ^2^ Department of Pharmacology, Naval Medical University, Shanghai, China

**Keywords:** endogenous protective factors, analogues, diabetes mellitus, atherosclerosis, therapy

## Abstract

The complications of macrovascular atherosclerosis are the leading cause of disability and mortality in patients with diabetes. It is generally believed that the pathogenesis of diabetic vascular complications is initiated by the imbalance between injury and endogenous protective factors. Multiple endogenous protective factors secreted by endothelium, liver, skeletal muscle and other tissues are recognized of their importance in combating injury factors and maintaining the homeostasis of vasculatures in diabetes. Among them, glucagon-like peptide-1 based drugs were clinically proven to be effective and recommended as the first-line medicine for the treatment of type 2 diabetic patients with high risks or established arteriosclerotic cardiovascular disease (CVD). Some molecules such as irisin and lipoxins have recently been perceived as new protective factors on diabetic atherosclerosis, while the protective role of HDL has been reinterpreted since the failure of several clinical trials to raise HDL therapy on cardiovascular events. The current review aims to summarize systemic endogenous protective factors for diabetes-associated atherosclerosis and discuss their mechanisms and potential therapeutic strategy or their analogues. In particular, we focus on the existing barriers or obstacles that need to be overcome in developing new therapeutic approaches for macrovascular complications of diabetes.

## Introduction

Atherosclerosis-related vascular complications are the main cause of reduced life quality and expectancy in diabetics. Atherosclerosis is known as chronic inflammatory diseases involving a variety of cells and pathogenesis, which is characterized by endothelial dysfunction, foam cell formation, the accumulation of lipids and eventually leads to lesion development (1). In diabetes, hyperglycemia is one of the main causes of atherosclerosis, but it is often combined with other risk factors, namely, dyslipidemia and hypertension to aggravate vascular injury synergistically. It is indistinguishable at histological examination of atherosclerotic lesions in patients with hyperglycemia to those with other risk factors like hypercholesterolemia or smoking (2). However, as reported, diabetic patients are four to five times more likely to develop cardiovascular diseases or stroke than individuals without diabetes (3). This obvious discrepancy may be owing to unique pathophysiological mechanisms of diabetes-associated atherosclerosis. It is generally believed that the pathogenesis of diabetic vascular complications is initiated by the imbalance between injury and endogenous protective factors. Among them, multiple mechanisms of destructive factors have been extensively studied to mediate the adverse effects on vascular tissues of hyperglycemia (4, 5). These include overproduction of reactive oxygen species (ROS) (6), formation of advanced glycation end products (AGEs) (7), activation of proinflammatory pathways, and increased expression of adhesion molecules. By contrast, very few studies have focused on the endogenous protective factors that exist to neutralize toxic AGEs, oxidative stress, and inflammation actions. The 50-Year Medalist Study showed diabetic patients with long-term poor glycemic did not correlate with vascular complications, and the effects of strict blood glucose control on cardiovascular disease (CVD) are marginal, suggesting the existence of endogenous protective factors can neutralize toxic effects of hyperglycemia and counteract mechanisms responsible for complication progression (8, 9). However, to our knowledge, most of these studies and reviews focus on protective factors on microvascular complications especially diabetic retinopathy and diabetic nephropathy, while systemic reviews update about endogenous protective factors on diabetic atherosclerosis or macrovascular complications are relatively lacked. Herein, we review the endogenous protective factors and potential therapeutic analogues that were proven to be effective at least in animal models with diabetic atherosclerosis.

### NO and eNOS

Endothelial dysfunction, characterized by the lowered bioavailability of nitric oxide (NO), is recognized to be the first step of atherosclerosis and cardiovascular disease (10). Oxidative stress or increased ROS formation in the vascular wall is a significant driver to reduce bioactive NO in underpinning diabetic vascular complications (11). Endothelial NO protects against atherosclerosis by mediating vasodilation, inhibiting platelet adhesion, leukocyte chemotaxis, and cellular proliferation of vascular smooth muscle cells, thus promoting endothelial cell barrier integrity (12). Atheroprotective NO is mainly produced by enzyme endothelial nitric oxide synthase (eNOS), which is a dimeric NOS isoform specifically expressed in endothelial cells and known as an endothelial protective factor in atherosclerosis while the inducible nitric oxide synthase (iNOS), another NOS isoform induced by cytokines and other agents expressed in almost any cell type, shown to be proatherogenic (13). eNOS is constitutively expressed in the caveolae and maintains its basal activity by interacting with Caveolin-1 (Cav-1), the main coat protein of caveolae. The regulation of eNOS is much complicated in atherosclerosis. On one hand, eNOS can be activated by phosphorylation of the enzyme response to various factors, such as increased shear stress or insulin stimulation, then coupled with cofactor (BH4) or substrate (L-arginine), leading to the production of protective NO. On the other hand, uncoupling eNOS in disease settings can be a source of superoxide, resulting in NO inactivation. In diabetes and its related atherosclerosis, hyperglycemia negatively regulated eNOS phosphorylation, causing eNOS uncoupling and reduced bioactive NO by increasing AGEs formation and activating Protein kinase C (PKC) pathway (14–16). Thus, the NO bioavailability depends on the expression level of eNOS, but more importantly, the eNOS activity.

Multiple conventional drugs such as statins or angiotension converting enzyme inhibitors (ACEi) can reduce vascular oxidative stress and increase bioactive NO in clinical or preclinical settings, but it remains elusive because all these drugs are pleiotropic or secondary effects rather than direct regulation of eNOS derived NO. CavNOxin is a Cav-1-derived peptide with T90, 91, F92 substituted to alanines. It has been identified to highly specific increase eNOS activity by preventing eNOS uncoupling (17, 18). As reported, CavNOxin could attenuate total aortic plaque up to 70% in diabetic apolipoprotein E knockout (ApoE^−/−^) mice, a well-established model of experimental atherosclerosis, whereas mice lacking eNOS show resistance to CavNOxin treatment, suggesting endogenous eNOS activation can provide atheroprotection in diabetes (12). Beyond that, there are amounts of other small molecules, such as compounds AVE9488, AVE3085 and trans-resveratrol, enhancing eNOS expression and preventing eNOS uncoupling under pathophysiological conditions and also showing therapeutic potential *in vitro* studies (13). AVE9488 and AVE3085 were known as novel eNOS transcription enhancers. AVE9488 enhanced vascular content of the essential eNOS cofactor BH_4_ and reversed eNOS uncoupling (19). Long-term treatment with AVE9488 improved cardiac remodeling and protected ischemia-reperfusion damage through increasing NO bioavailability (20). AVE3085 prevented endothelial dysfunction in arteries by regulating the expression of eNOS at different phosphorylation sites and also inhibition of arginase and iNOS (21). In addition, trans-resveratrol, a class of flavonoid compounds, has been demonstrated to increase endothelial NO production through diverse mechanisms, namely, upregulating of eNOS expression, stimulating of eNOS enzymatic activity, and preventing of eNOS uncoupling (22). Pharmacological interventions of them regulated eNOS/NO signaling pathway mainly through eNOS phosphorylation and protein-interactions. In this context, further in-depth studies are required to have a better understanding of how to improve eNOS-derived NO in patients with diabetes.

### Lipoxins

Growing evidence suggests that chronic inflammation plays an important role in the pathophysiology of diabetes and diabetes-related vascular complications, therefore, the endogenous proresolution molecules and synthetic analogs targeting inflammation resolution are increasingly recognized as a therapeutic strategy to ameliorate diabetes, prevent its progression and vascular complications (23–26).

The omega-6 arachidonic acid (AA)-derived lipoxins [LXs], namely, LXA4 and LXB4 in mammals, are the first recognized endogenous lipid mediators that have dual anti-inflammatory and pro-resolution activities (27). They are produced by different biosynthetic pathways, involving the interaction of activated neutrophils within the epithelium, endothelium, and platelets (28).

Previous clinical data have shown that circulating levels of LXs or arachidonic acid (AA) are reduced in patients with obesity (29), diabetes and its complications (30), suggesting LXs maybe protective factors in metabolic disease and associated vascular complications. Borgesön et al. (31) reported that LXA4 and a benzo-LXA4 analogue reduced obesity-induced adipose inflammation by promoting a macrophage M1-to-M2 switch, modulating adipose autophagy. They demonstrated the Lipoxin-mediated protection was independent of adiponectin by using adiponectin^−/−^ mice.

Recently, Brennan (32) reported that LXs could prevent and attenuate the development of atherosclerotic lesions in diabetic ApoE^−/−^ mice but not in nondiabetic ApoE^−/−^ mice. The mechanism involved the inhibition of the vascular smooth muscle cell proliferation and endothelial cell inflammation. They showed that metabolic parameters were not changed by LXs, suggesting that LXs-mediated protection was independent of glycemic control. Consistently with the animal experiments, LXA4 suppressed inflammatory cytokine release, namely, tumor necrosis factor-α and interleukin-1β in human carotid plaque explants. These data suggest that LX and its analogue therapy may offer a novel therapeutic approach in the context of diabetes-associated vascular complications (33).

### Adiponectin

Adiponectin is a widely studied adipokine with anti-inflammatory, antioxidant, antiatherogenic, and insulin-sensitizing properties (34–36). Adiponectin exerts its biological role mainly by binding to its specific receptors, namely, adiponectin receptor 1 (AdipoR1), adiponectin receptor 2 (AdipoR2), and newly discovered T-cadherin (37). The receptors are abundantly expressed in cardiomyocytes, vascular smooth muscle cells, and endothelial cells and were supposed to be involved in atherosclerosis development (38–40).

Clinically, a large number of epidemiological studies suggested that the level of serum adiponectin in patients with obesity, type 2 diabetes, and atherosclerotic cardiovascular disease were significantly lower than that in normal subjects (41–43), while the low calorie diets, physical exercise, and bariatric surgery leading to weight loss may result in consistent increases of adiponectin levels (44). It was proved that hypoadiponectinemia could predict endothelial dysfunction in healthy men (45) and predict atherosclerosis in patients with end-stage renal disease (46). Further, adiponectin-deficient mice showed significantly increased neointimal thickening disordered endothelium-dependent vasodilation compared with wild-type mice (47–49). Moreover, adiponectin overexpression in the ApoE^−/−^ mouse, can reduce the progression of fatty streak lesion through attenuating endothelial inflammatory response and macrophage to foam cell transformation (50). Based above, adiponectin is proposed as a predictive factor and a potential therapeutic target for atherosclerotic cardiovascular disease.

Adiponectin is known to exert vasoprotective actions through several mechanisms (51). A number of studies show that adiponectin could suppress the activation of pro-inflammatory and adhesion molecules, inhibit the monocyte/macrophage migration to the vascular wall and prevent the formation of foam cells (52–54). *In vitro* and *in vivo* studies indicated that adiponectin also reduced oxidative stress and high glucose-induced apoptosis, protected against endothelial dysfunction induced by OxLDL (55, 56). Furthermore, adiponectin can inhibit several atherogenic growth factors including platelet-derived growth factor to block the proliferation and migration of human aortic smooth muscle cells (57). In addition, adiponectin exerts the vascular protective function by directly enhancing the eNOS activity and improving the NO production depending on AdipoR1–AMPK signaling pathways (53, 58).

Several drugs (e.g., thiazolidinediones, angiotensin receptor blocker, sodium glucose cotransporter 2 inhibitors and incretins) have an effective influence on circulating adiponectin level through multiple mechanisms such as transcription regulation of adiponectin expression and pathways that enhance adipogenesis and insulin sensitivity (51). However, the actual clinical application of exogenous recombinant adiponectin is scarce due to the complexity of adiponectin multimers structure and its short half-life *in vivo* (59), and designing agonists to activate adiponectin receptor is suggested as an alternative strategy to maximize the beneficial effects of adiponectin. ADP355 and osmotin are two adipoR agonists among the numerous promising candidates in preclinical development. ADP355, an adiponectin-derived active peptide, was reported to ameliorate lipid metabolism and inhibit atherosclerosis in apoE^−/−^ mice (60). Osmotin, an adiponectin homolog, that was found to function as an agonist for AdipoR1 (61) and infusion of osmotin could suppress the development of aortic atherosclerotic lesions in apoE^−/−^ mice (62). In comparison, AdipoRon is a selective, orally active, synthetic small-molecule agonist, which can bind and activate AdipoR1 and AdipoR2, attenuated insulin resistance and glucose intolerance, improving lipid metabolism in high-fat diet mice (63). Oral administration of AdipoRon in C57BL/6J mice significantly suppressed arterial injury-induced neointimal hyperplasia by targeting VSMC proliferative signaling events (64), but there are lacking studies up to now to examine the role of AdipoRon on atherosclerosis in diabetes models. Further studies are needed to evaluate the clinical implications targeting to adiponectin or its receptors in the treatment of cardiometabolic diseases in diabetes.

### Omentin

Omentin (also known as omentin-1 or intelectin-1) is a newly discovered adipokine with insulin-sensitizing, antioxidant, anti-inflammatory, and anti-atherosclerotic effects (65). It is preferentially secreted from the visceral fat stromal vascular cells, and also less expressed in endothelial cells, lung, heart, and placenta (66).

Recently, the level of omentin-1 was considered as a new biomarker of vascular endothelial function, especially for diabetic patients (67). Several cross-sectional studies reported that the concentration of omentin-1 decreased in patients with type 2 diabetes (T2DM) (68), coronary artery disease (CAD) (69, 70) or obese individuals with higher cardiovascular risk (71). Circulating omentin-1 level are negatively correlated with carotid intima-media thickness (IMT), arterial stiffness and carotid plaque in healthy men and type 2 diabetic patient (68, 72). Consistently, in apolipoprotein E-deficient mice, omentin exhibited a significant reduction of the atherosclerotic areas by affecting the phenotypes of macrophages (73). On the contrary, a recent population-based cohort studies showed higher omentin concentrations were associated with a higher risk of primary cardiovascular events in diabetic patients even after adjusting for other cardiovascular risk factors including adiponectin (66). It appears possible that this association reflects a counterregulatory mechanism.

Liu et al. (74) found that omentin-1 protected against high glucose-induced vascular-endothelial dysfunction through its ability to inhibit reactive oxygen species (ROS) and increase NO production *via* activation of eNOS signaling pathway in isolated mouse aortas and mouse aortic endothelial cells (MAECs). Another evidence from diabetic rat studies indicated the protective effects of omentin against endothelial dysfunction through its actions on anti-inflammatory and antioxidant in perivascular adipose tissue (65). These studies suggested targeting circulating omentin levels may present therapeutic potential for cardiovascular diseases in diabetic patients.

### HDLs and apoA-Ⅰ

High-density lipoprotein cholesterol (HDLs) are complex polymolecular assemblies produced by the liver, jejunum and in serum. They are consisting of a hydrophobic lipid core (TGs and cholesterols) and an outer layer of phospholipids and apolipoproteins (mainly apoA-I), which facilitate reverse cholesterol transport (RCT) from peripheral tissues to liver.

In the past few decades, HDLs are recognized as a protective factor against vascular complications with diabetes mellitus (DM) due to its multiple functions encompassing anti-inflammatory, anti-oxidative, anti-thrombotic, and anti-diabetic properties (75, 76). A wealth of epidemiological and clinical studies indicated low HDL levels are independent risk factors for the development of atherosclerotic CVD or stroke with DM (77, 78). Similarly, alterations in plasma HDL and its related factors, LDL-C/HDL-C and TC/HDL-C ratio, showed a potential value in predicting glycemic control or cardiovascular function in diabetic patients (79, 80). A less favorable lipid profile could explain the success of lipid-modifying therapies, such as statins, in reducing adverse cardiovascular events. However, until now, HDL is still not considered a primary target of therapy in the latest national clinical guidelines on cholesterol management (81, 82). Although deficiency (83, 84) or overexpressing (85, 86) of high density lipoprotein or apolipoprotein A-I has clearly demonstrated a reduction or acceleration of atherosclerosis respectively in mice, several clinical studies aiming to raise HDL level therapies like CETP inhibitors or niacin have no significant benefits to cardiovascular events in patients with or without DM (87). An international double-blind randomized clinical showed infusion of recombinant HDL or apoA-I fail to regress plaque in coronary arteries of patients with acute coronary syndrome (88). One possible explanation of these negative results is that biological HDL could be adversely modified to be “dysfunctional HDL” by diabetes and atherosclerosis through the alteration of specific components and modifications of oxidation or glycation of HDL particles. This was supported by previous studies that HDL particle size and the distribution of HDL sub-classes were significantly altered in patients with coronary heart disease (CAD) complicated by DM compared with those in CAD without DM (89). Clinical data showed that highly elevated HDL did not always protect against cardiovascular disease, sometimes even diametrically opposed (90). Moreover, measures of HDL function such as cholesterol efflux capacity from macrophages is more effective in predicting the prevalence and incidence of CVD than measuring quantity of HDL cholesterol or apoA-I (91, 92). These results suggested that future development of novel therapies aiming HDL should focus on overcoming HDL dysfunction rather than improving the quantity of HDL. Indeed, development of HDL analogues and apoA-I mimetic peptides in view of overcoming the limits of the low efficiency of HDL in these processes do show some promise. Some novel apoA-I mimetic peptide, such as D-4F (93) and P12 (94), were believed to suppress atherosclerosis by promoting physiological HDL function *in vitro* studies or a murine model of diabetic atherosclerosis. Further clinical studies involving these compounds on vascular complications in diabetic patients are eagerly awaited.

### Incretins (GLP-1 and GIP)

Incretins are a family of gut-derived peptide hormones which include glucagon-like peptide-1 (GLP-1) and glucose-dependent insulinotropic polypeptide (GIP) (95), which are respectively secreted from L cells of the distal intestine and the K cells of the proximal intestine in response to ingestions of various nutrients. They both stimulate insulin secretion in a glucose-dependent manner by binding with specific receptors, namely GLP-1 receptors (GLP-1R) and GIP receptors (GIPR) on β-pancreatic cells (96, 97).

In diabetes, the secretion of incretins, especially the GLP-1 after meal ingestion were significantly reduced compared with healthy individuals. Targeting this deficiency by using GLP-1-based drugs is a well-established approach in T2DM. Since GLP-1 is easily degraded by dipeptidyl peptidase-4 (DPP-4), DPP-resistant GLP-1 receptor agonists (GLP-1 RAs) were designed basing on either exendin-4 (drugs such as exenatide and lixisenatide) or human GLP-1 (drugs such as liraglutide, dulaglutide, and semaglutide), and therefore, have a prolonged half-life. DPP-4 inhibitors such as sitagliptin and sagliptin are also effective strategies by increasing the concentration of endogenous GLP-1. GLP-1 RAs exert glucoregulatory effects *via* glucose-dependent secretion of insulin, inhibition of glucagon release. Further, the presence of GLP-1R has been detected in a wide range of organs, namely, vessels, heart, brain, and gastrointestinal tract (98, 99). The extrapancreatic actions of GLP-1 include inhibition of gastric emptying, gastric acid secretion, and suppressing appetite, thereby fulfilling the definition of GLP-1 as an enterogastrone. GLP-1 RAs have been effective glucose-lowering drugs for a decade with weight loss, lower risk of hypoglycemia, and even cardiovascular benefits.

Indeed, numerous clinical studies have shown the cardiovascular protective effects of GLP-1 RAs on atherosclerosis, coronary arterial disease (CAD), and cerebrovascular disease (100). For example, in an ApoE^−/−^ mouse model, liraglutide has shown to inhibit atherosclerotic plaque formation and enhanced plaque stability and endothelial function (101). In T2DM patients, Rizzo and colleagues (102) reported 8 months treatment of liraglutide therapy leads to a reduction in carotid intima media thickness (cIMT), a surrogate marker for CVD risk, and this effete is independently of its effect on plasma glucose and lipids concentrations. In the cardiovascular outcomes trials of liraglutide (the LEADER study), liraglutide could further significantly reduce the risk of major cardiovascular adverse events by 13% in patients already received cardiovascular secondary prevention drugs (103). In these studies, the cardiovascular protective effects of GLP-1 RAs was independent of glycemic control (104, 105).

GLP-1/GLP-1RAs may mediate effects on cardiovascular outcomes through effects on other risk factors such as the decreasing blood pressure values, weight reduction and improvement of dyslipidemia and endothelial dysfunction. Accumulating evidence suggests that GLP-1/GLP-1RAs increases the production of endothelial nitric oxide (NO) (106), reduces endothelial dysfunction (107), inflammation and oxidative stress (108) and also inhibits the transformation from monocytes to foam cells (109). In addition, treatment with GLP-1RA also increased circulating adiponectin levels (110), which play a protective role in the cardiovascular system. Based on these findings and mechanisms, GLP-1RAs have now been recommended by the ESC/EASD (European Society of Cardiology/European Association for the Study of Diabetes) released guidelines as one of the first-line therapies in type 2 diabetic patients with high risks or established cardiovascular disease (111, 112).

Compared with GLP-1, no GIP receptor agonist is utilized clinically to date because the glucoregulatory effects of GIP shows to be weakened in individuals with diabetes (113, 114). In patients with hyperglycemia and liver cirrhosis, GIP can stimulate the secretion of glucagon, resulting in increased glucose levels (115, 116). Inhibition of physiological GIP has been shown to alleviate obesity and insulin resistance under high-fat diet conditions (117). Consistently, GIPR antagonist can enhance insulin sensitivity, improve glucose tolerance, and reduce weight gain (118, 119). These studies indicated that the GIP treatment might increase the risk of metabolic deterioration in diabetes. However, the concern for its safety leading the neglection for its function on cardiovascular health. Recently, infusion of GIP in pharmacological dose have been found to prevent accumulation of aortic plaque, macrophages and foam cells in diabetic apolipoprotein E-null mice (120). Anti-atherosclerosis function of GIP may lie in the mechanism of improving NO production in VECs and activation of AMPK, inhibiting cell proliferation in VSMCs, and inhibiting inflammatory effect of monocytes, macrophages, and adipocytes (121). These observations suggest that GIP under pharmacological concentration may induce anti-diabetic and antiatherogenic effects. More fundamental studies and preclinical trials such as dual agonists targeting for GIPR and GLP-1R are still ongoing.

### L-Carnosine

L-carnosine (beta-alanyl-L-histidine) is an endogenous dipeptide composed of β-alanine and l-histidine and highly expressed in skeletal muscle, brain and less in cardiac muscles (122). It is synthesized endogenously by carnosine synthase in skeletal muscle cells, glial cells, and myocytes, and it also could be obtained from dietary sources such as meat and fish (123). L-carnosine is a quencher of the Reactive Carbonyl Species (RCS), which are derived from Advanced Glycation (AGEs) and lipoxidation end-products. Therefore, it not only plays a major role in inhibiting AGEs formation, but also prevents the activation of pro-oxidative and pro-inflammatory pathways secondary to its ability to trap RCS (124, 125).

Several studies have been conducted on the effect of carnosine on diabetes and its complications, thanks to its inhibitory effect on the production of AGEs and oxygen toxicity. As expected, this endogenous dipeptide is proved to reduce cholesterol and triglyceride levels and ameliorate the dyslipidemic blood profile in multiple animal models, namely, diabetic Balb/cA mice (126), finishing Pigs (127), and obese Zucker rats (128). Further, Brown et al. (122) reported L-carnosine supplementation in drinking water for 20 weeks reduced plasma triglycerides, changed plaque atherosclerotic composition, and suppressed atherosclerotic plaque instability in diabetic ApoE^−/−^ mice. Consistently, *in vitro* studies revealed that L-carnosine was able to inhibit glycation of low-density lipoproteins and reduce the formation of foam cells when incubated with glycated LDLs (129). These findings may partly explain the modifications in plaque composition observed by Brown et al. However, since L-carnosine is rapidly inactivated by serum carnosinase in human, the search for carnosine derivatives that are resistant to hydrolysis by carnosinase enzymes maybe a more suitable strategy. Stefano Menini and his colleagues (130) showed the diabetic ApoE^−/−^ mice treated with D-carnosine-octylester (DCO), a bioavailable pro-drug of the carnosinase-resistant D-carnosine, for 20 weeks resulted in a more stable plaque phenotype, and even further a reduced atherosclerotic lesion size compared to untreated animals. In more detail, DCO treatment for 11 weeks also afforded partial protection from diabetes-induced atherosclerosis. Interestingly, the protective effect of DCO was more effectively achieved by early treatment (treated with DCO from weeks 1 to 11) than by late treatment (treated with DCO from weeks 9 to 19) due to early inhibition of AGE formation. The phenotypes obtained by carnosine and DCO is regardless of lipidemic and glycemic status, suggesting the protective effect is independent of hypoglycemic and lipid-lowering effect. They also showed the molecular mechanisms underlying the protective effects by DCO was associated with reduced foam cell accumulation, inflammation and apoptosis and also with increased content of collagen and smooth muscle cells.

In human study, supplementation with a daily dose of 2 g carnosine improved glucose metabolism, preserved insulin sensitivity and secretion in overweight and obese individuals (131). There is an ongoing randomized controlled trial (RCT) focusing on carnosine on cardiometabolic health and cognitive function in patients with prediabetes and type 2 diabetes (132). If this trial proves to be effective, more well-designed clinical trials with larger samples are needed to confirm the potential roles of carnosine and its derivatives in the prevention and treatment of diabetes and diabetic cardiovascular disease.

### Irisin

Irisin is a recently recognized cytokine that is produced by plasma membrane protein fibronectin type III domain-containing protein 5 (FNDC5) cleavage. It is mainly secreted by skeletal muscle and released into the blood circulation during exercise, and known as a mediator for browning of subcutaneous white adipose tissue (WAT) and increased thermogenesis and alleviate insulin resistance (133, 134).

There have been a lot of studies investigating the association between circulating irisin with obesity (135–138) and diabetes mellitus (136, 139–145). Majority of studies in human and animals showed that lower circulating levels of irisin were associated with obesity (135, 137) and T2DM (136, 140–145), but so far with inconsistent and controversial results; the opposite trend was also found in subjects with obesity (136, 138), metabolic syndrome (146), and T2DM (147). It is still controversial whether disease condition increases or decreases circulating irisin levels. These discrepant findings may be due to the difference of study population, type of disease, and experimental design. Elevated irisin levels in those patients may act as a compensatory mechanism to combat metabolic disorders.

In contrast, not many, but consistent results show that decreased plasma levels of irisin are independently associated with endothelial dysfunction (137), flow-mediated arterial dilation (141) and presence and severity of coronary artery disease (CAD) (148), implying irisin may ameliorate vascular endothelial dysfunction and treating atherosclerosis, and this is also an explanation for the effective role of physical exercise in the prevention and management of cardiometabolic risk and in the treatment of metabolic syndrome and its complications. A recent animal study has also suggested that irisin treatment suppressed endothelial injury and reduced the degree of aortic atherosclerotic plaque in apolipoprotein E-knock out diabetic mice (149), suggesting irisin could be therapeutic for atherosclerotic vascular diseases in diabetes. Consistently, a case–control study from Egypt showed irisin was a reliable diagnostic or prognostic biomarker for atherosclerosis in type 2 diabetic female patients.

Experiments *in vivo* and *in vitro* indicated that the pathophysiological mechanism of endothelium-protective action of irisin may involve activation of the AMPK–PI3K–Akt–eNOS signaling pathway (149), inhibiting AGEs-induced oxidative stress and NLRP3 inflammasome signaling (150), promoting endothelial cell proliferation (151). Taken together, it has been revealed that irisin level played a beneficial role on metabolic diseases and related vascular complications, but more studies are still needed to prove it to be a therapeutic for atherosclerotic vascular diseases in diabetes mellitus.

## Conclusion

The imbalance between injury and endogenous protective factors was thought of as an initiating pathogenesis contributing to diabetic vascular complications. In most cases, the serum levels of protective factors were significantly reduced under diabetic condition and diabetic macrovascular complication as shown in **Table 1**, representing a potential to serve as diagnostic or prognostic biomarkers of cardiovascular complications in diabetic patients.

**Table 1:** A list of endogenous protective factors and their serum level under diabetic condition and its macrovascular complication.

Endogenous protective factors	Serum level under diabetic condition	Serum level under diabetic macrovascular complication	Production/expression site
eNOS activity	↓	↓	endothelial cells
Adiponectin	↓	↓	adipose tissue
Omentin	↓	↓/↑	adipose tissue
HDLs/apoA-I	↓	↓	liver, jejunum and in serum
GLP-1	↓	↓	L cells of the distal intestine
Lipoxins	↓	↓	epithelium, endothelium, and platelets
L-carnosine^a^	–	–	skeletal muscle, brain, cardiac muscles
Irisin	↓/↑	↓	skeletal muscle and released into serum

–, unknown; ↓, decreased; ↑, increased; ↓/↑, decreased or increased results were observed with controversy.

a L-carnosine is rapidly inactivated by serum carnosinase in human.

**Figure 1:**
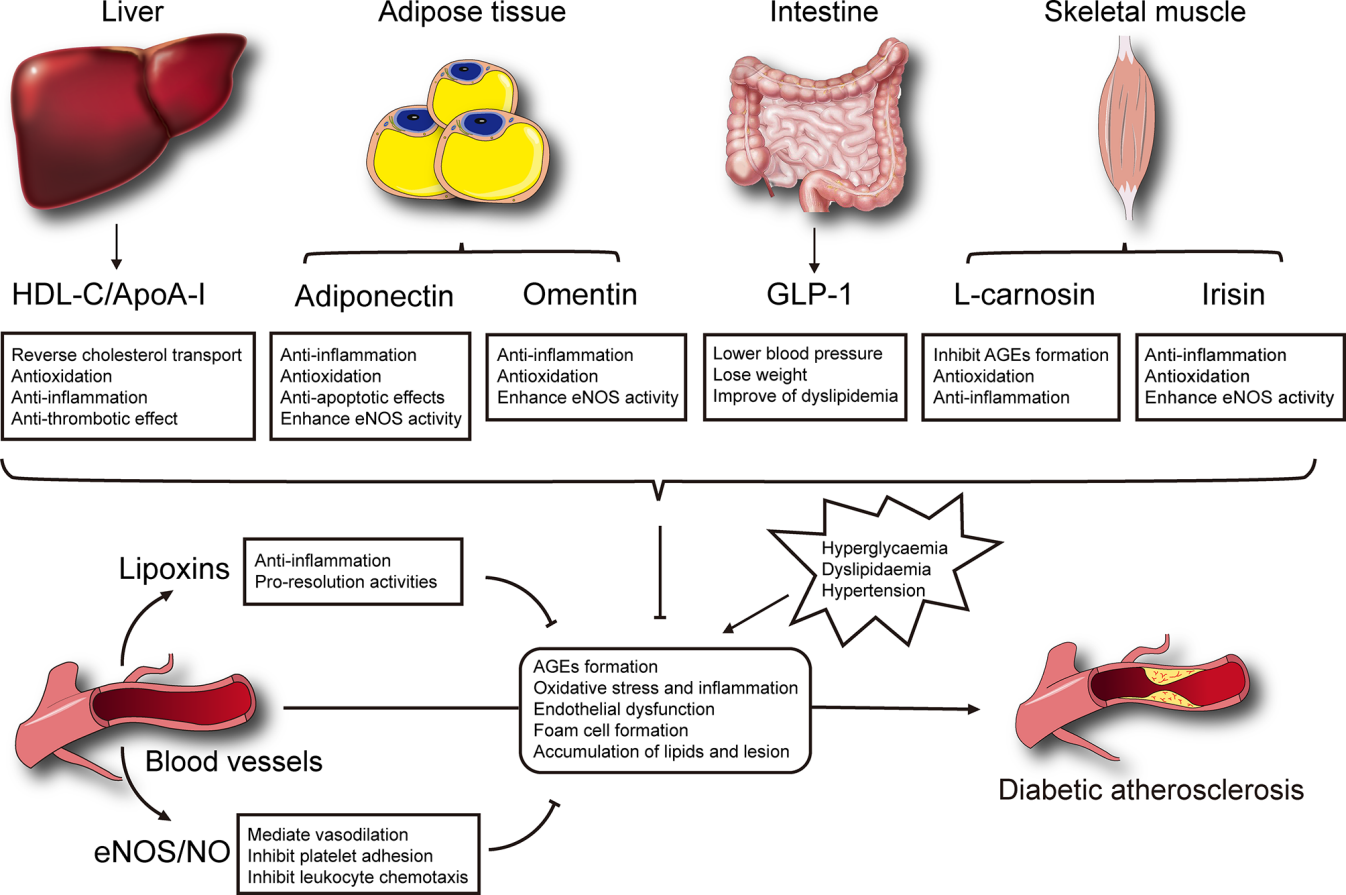
Selected mechanisms of endogenous protective factors on the development of diabetic atherosclerosis.

Currently, few effective therapeutic methods are available for the management of diabetic macrovascular complications. The presence of endogenous protective factors secreted by endothelium, liver and other tissues could alleviate development and progression of diabetic atherosclerosis through multiple mechanisms (**Figure 1**). Further clinical therapeutics targeting to enhancing protective factors showed a new promising opportunity in preventing or delaying the vascular complications of diabetes. Incretin mimetics (GLP-1RAs) were convinced significantly of reducing the major cardiovascular adverse events, and recommended as the first-line medicine in type 2 diabetes mellitus patients with cardiovascular risk factors. In contrast, several large-scale clinical trials aiming to raise HDL cholesterol in cohorts fail to show benefits in cardiovascular events. It seems to be a solution to develop novel analogues or mimetic peptides based on function rather quantity. Moreover, adipokines such as adiponectin and omentin, and myokines such as irisin are also providing a new perspective for understanding the development of diabetic complications and representing promising therapeutic prospects. A note of caution is that the therapeutic effects of these factors were obtained in preclinical evidence, thus, human studies with large quantity and high quality are required to validate the results to the clinical situation.

## Author Contributions

Both CW and JC equally contributed to writing the manuscript and sourcing references for the review. PW and SQ contributed to discussions and editing of the manuscript. WL and JL conceived the outline of this paper and participated in critical review and further revision of the manuscript. All authors listed have made a substantial, direct, and intellectual contribution to the work and approved it for publication.

## Funding

This research was supported by research project on aging, maternal and child health from the Shanghai Health Commission (2020YJZX0131) and medical innovation research project from the Shanghai Science and Technology Committee (20Y11905200).

## Conflict of Interest

The authors declare that the research was conducted in the absence of any commercial or financial relationships that could be construed as a potential conflict of interest.

## Publisher’s Note

All claims expressed in this article are solely those of the authors and do not necessarily represent those of their affiliated organizations, or those of the publisher, the editors and the reviewers. Any product that may be evaluated in this article, or claim that may be made by its manufacturer, is not guaranteed or endorsed by the publisher.
